# Complete Genome Sequence of *Oceanotoga* sp. Strain T3B (DSM 15011)

**DOI:** 10.1128/mra.00022-23

**Published:** 2023-03-01

**Authors:** Anne A. Farrell, Olga Zhaxybayeva, Camilla L. Nesbø

**Affiliations:** a Department of Biological Sciences, Dartmouth College, Hanover, New Hampshire, USA; b Department of Computer Science, Dartmouth College, Hanover, New Hampshire, USA; c Department of Biological Sciences, University of Alberta, Edmonton, Alberta, Canada; d Department of Chemical Engineering and Applied Chemistry, University of Toronto, Toronto, Ontario, Canada; University of Delaware College of Engineering

## Abstract

*Oceanotoga* sp. strain T3B was isolated from an estuarine sinkhole in the Bahamas. Here, we report its complete genome, which is currently the only sequenced genome from the genus *Oceanotoga*. The genome sequence provides new data for the genus *Oceanotoga*.

## ANNOUNCEMENT

**FIG 1:**
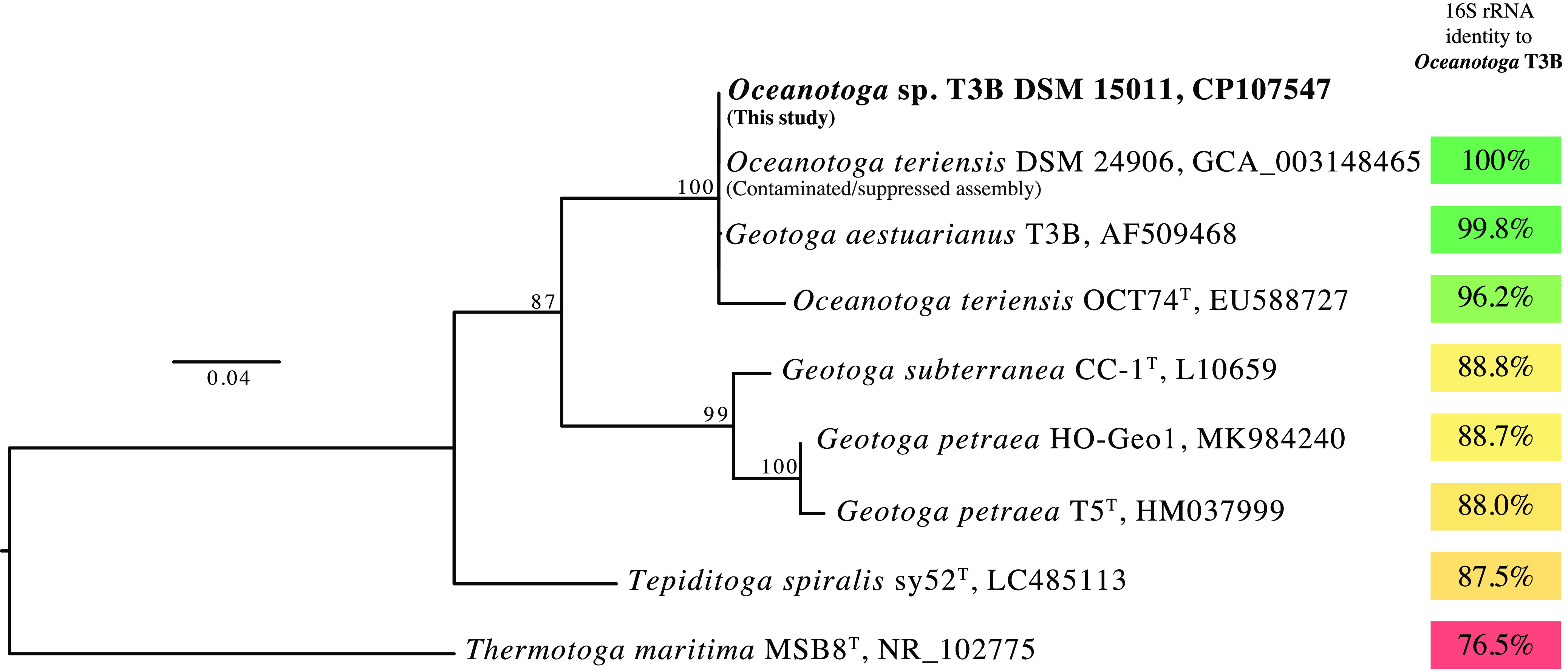
Phylogenetic relationships and sequence identity among 16S rRNA genes from taxa closely related to *Oceanotoga* sp. strain T3B. The 16S rRNA gene sequence from this project (GenBank accession number CP107547) was aligned with the 16S rRNA gene sequence originally deposited as the Geotoga aestuarianus T3B sequence (GenBank accession number AF509468), as well as with 16S rRNA gene sequences from all type strains and strains with sequenced genomes from *Geotoga*, *Oceanotoga*, and *Tepiditoga*, using MAFFT v.7.305b (9). The pairwise sequence identity values were calculated by extracting two sequences from the multiple-sequence alignment, removing shared gaps and unaligned sequence ends, and calculating the sequence identity of the remaining alignment using in-house scripts. The maximum likelihood tree was reconstructed in IQ-TREE v.1.6.12 (10) with the TIM3+F+G4 substitution model (11). Support of the topology was obtained from 100 bootstrap pseudosamples. The tree was rooted with Thermotoga maritima MSB8^T^. Type strains are indicated by a superscript T. GenBank accession numbers are listed next to the strain names.

*Oceanotoga* sp. strain T3B was isolated from an estuarian karst sinkhole in Stafford Creek, Bahamas (1). In 2002, its 16S rRNA gene sequence was deposited in GenBank as Geotoga aestuarianus T3B (GenBank accession number AF509468), but the strain has not been validly described. However, the sequence of GenBank accession number AF509468 is more closely related to the 16S rRNA gene sequence of the only described species of *Oceanotoga* (2) than to the two described *Geotoga* species (3) (Fig. 1). A genome assembly for Oceanotoga teriensis DSM 24906 (GenBank accession number GCA_003148465) is marked as contaminated and suppressed. We sequenced the genome of strain T3B to increase genomic representation for the *Oceanotoga* genus.

*Oceanotoga* sp. strain T3B was acquired from the DSMZ Culture Collection (DSM identification number 15011) and cultivated at 37°C for 48 h in DSM medium 1163a under anaerobic conditions (N_2_ atmosphere). Total DNA was extracted using the Qiagen MagAttract high-molecular-weight (HMW) DNA kit, following the protocol for Gram-positive bacteria. The purified DNA was sequenced, without shearing or size selection, on a MinION system (Oxford Nanopore Technologies [ONT]) with a Flongle flow cell (FLO-FLG001; ONT) using the ligation sequencing kit SQK-LSK109 (ONT). This procedure produced around 320,000 reads, with an estimated *N*_50_ value of 6.98 kb and a total size of 716.36 Mb. Base calling using Guppy v.6.1.5 (4) produced 590.97 Mb with Q scores of ≥9; the average score was 13.83.

The reads were assembled using Flye v.283 (5) (all reads, reads of >1,000 bp, or reads of >5,000 bp) and Canu v.2.2 (6) (all reads). All assemblies resulted in one circular genome of 2.8 Mb. The final genome was produced by manual curation of the Flye assembly from all reads. Errors in homopolymer regions were corrected manually by using MAUVE alignments (7) of all assemblies and by mapping trimmed reads from Canu. The genome was annotated using the NCBI Prokaryotic Genome Annotation Pipeline (PGAP) (8).

The closed genome is 2,841,299 bp and has a GC content of 26.2%. The average sequencing depth is 206.0×. The genome has 2,731 genes, including 2,657 protein-coding genes, 50 tRNAs, and 21 rRNAs distributed across 7 rRNA operons. The genome also contains 8 CRISPR regions.

The newly sequenced genome has >99% average nucleotide identity (ANI) and 100% 16S rRNA gene identity to the suppressed genome of Oceanotoga teriensis DSM 24906 (GenBank accession number GCA_003148465) (Table 1 and Fig. 1). Therefore, we suspect that the suppressed genome record originates from T3B and not from an O. teriensis strain. Our circular, high-quality assembly for T3B replaces the draft genome from the inaccurate submission.

**TABLE 1:** ANI and AAI among *Oceanotoga*, *Geotoga*, and *Tepiditoga* strains with available genomes

Genome	ANI (%) (AAI [%])^*a*^					GenBank accession no.
	*Oceanotoga* sp. strain T3B (DSM 15011)	Oceanotoga teriensis DSM 24906	Geotoga petraea HO-Geo1	Geotoga petraea WG14	Tepiditoga spiralis sy52^T^	
*Oceanotoga* sp. strain T3B (DSM 15011)	100 (100)					CP107547
Oceanotoga teriensis DSM 24906 (contaminated/suppressed)	99.2 (99.0)	100 (100)				GCA_003148465
Geotoga petraea HO-Geo1	74.7 (59.2)	74.6 (59.3)	100 (100)			GCA_004768445
Geotoga petraea WG14	73.9 (59.4)	77.0 (59.9)	99.3 (98.8)	100 (100)		GCA_900102615
Tepiditoga spiralis sy52^T^	75.1 (57.7)	74.5 (57.4)	74.3 (56.8)	73.2 (56.7)	100 (100)	GCA_014701195

a The ANI and AAI values were calculated using the ANI/AAI matrix calculator (12).

Currently, only two genomes from the *Geotoga* genus, the sister genus of *Oceanotoga*, are available. Both genomes are from non-type strains of Geotoga petraea (GenBank accession numbers GCA_004768445 and GCA_900102615). The genome of T3B has <60% average amino acid identity (AAI) and <75% ANI with the two Geotoga petraea genomes (Table 1). These low genome-wide identities with *Geotoga*, in combination with the high sequence identity between the 16S rRNA genes of T3B and O. teriensis OCT74^T^ (Fig. 1), confirm that the T3B isolate indeed belongs to the *Oceanotoga* genus.

### Data availability.

All genome sequencing data were deposited in GenBank under BioProject accession number PRJNA889047, with SRA accession number SRR21862394, assembly accession number GCA_025837015, and nucleotide sequence accession number CP107547.
